# P‐Glycoprotein (P‐gp)/ABCB1 plays a functional role in extravillous trophoblast (EVT) invasion and is decreased in the pre‐eclamptic placenta

**DOI:** 10.1111/jcmm.13810

**Published:** 2018-09-05

**Authors:** Caroline E. Dunk, Jane J. Pappas, Phetcharawan Lye, Mark Kibschull, Mohsen Javam, Enrrico Bloise, Stephen J. Lye, Moshe Szyf, Stephen G. Matthews

**Affiliations:** ^1^ Lunenfeld Tanenbaum Research Institute Sinai Health System Toronto Canada; ^2^ Department of Pharmacology and Therapeutics McGill University Montreal Canada; ^3^ Department of Physiology University of Toronto Toronto Canada; ^4^ Department of Morphology Federal University of Minas Gerais Belo Horizonte Brazil; ^5^ Departments of Obstetrics and Gynecology and Medicine University of Toronto Toronto Canada

**Keywords:** ABCB1/P‐gp/MDR1, differentiation, invasion, placenta, pre‐eclampsia, trophoblast

## Abstract

Dysregulation of trophoblast differentiation is implicated in the placental pathologies of intrauterine growth restriction and pre‐eclampsia. P‐glycoprotein (P‐gp encoded by *ABCB1*) is an ATP‐binding cassette transporter present in the syncytiotrophoblast layer of the placenta where it acts as a molecular sieve. In this study, we show that P‐gp is also expressed in the proliferating cytotrophoblast (CT), the syncytiotrophoblast (ST) and the extravillous trophoblast (EVT), suggesting our hypothesis of a functional role for P‐gp in placental development. Silencing of *ABCB1,* via siRNA duplex, results in dramatically reduced invasion and migration, and increased tube formation and fusion in the EVT‐like HTR8/SV_neo_ cell line. In both EVT and CT explant differentiation experiments, silencing of *ABCB1* leads to induction of the fusion markers human hCG, ERVW‐1 and GJA1 and terminal differentiation of both trophoblast subtypes. Moreover, P‐gp protein levels are decreased in both the villous and the EVT of severe early‐onset pre‐eclamptic placentas. We conclude that, in addition to its role as a syncytial transporter, P‐gp is a key factor in the maintenance of both CT and EVT lineages and that its decrease in severe pre‐eclampsia may contribute to the syncytial and EVT placental pathologies associated with this disease.

## 1 INTRODUCTION

Pre‐eclampsia (PE) is a progressive and systemic vascular disorder that often leads to maternal and infant morbidity/mortality. Intrauterine growth restriction (IUGR) is a condition in which foetal growth and health are compromised. PE and IUGR are critical topics of health research as they affect a significant proportion of vulnerable populations (2%‐10% of all pregnant women worldwide^1^) and they remain extremely poorly understood.

“Severe early‐onset PE” is a distinct placental disease often compounded by IUGR, while “late‐onset PE” may be reflective of maternal inflammation and systemic endothelial disease.^2^ IUGR results from defective placental development and failure of the placenta to provide gas and nutritional exchanges to support foetal growth.^3^, ^4^ There is no proven treatment or prevention for PE. Low‐dose aspirin has been shown to have a protective effect,^5^ and blood tests to identify patients at high risk through elevated soluble FMS‐like tyrosine kinase‐1:placental growth factor ratios are now being used in certain populations with some success.^6^ With regard to IUGR, genetic factors have begun to be compiled.^7^

While little is known about PE and IUGR aetiology, placental pathology is known to be involved and may play a central role. The initiating cause of PE is thought to be the failure of the placental extravillous trophoblast (EVT) to effectively invade and physiologically transform the uterine spiral arteries, thereby decreasing the volume of maternal blood flow into the intervillous space while increasing blood pressure to a level often incompatible with normal pregnancy.^8^ There are several negative consequences of this decreased but high‐pressure intervillous blood flow, including oxidative and physiological stress in the villous placenta, disrupted villous trophoblast turnover and increased shedding of necrotic placental debris into the maternal circulation.^9^ While the molecular mechanisms underlying this failure are not well understood, an imbalance of trophoblast trophic factors in both PE and IUGR has been well documented.^10^

P‐glycoprotein (P‐gp, encoded by the *ABCB1* gene) is a critical component of the placental barrier that may have an important role in supporting the balance of trophic factors. P‐gp is an ATP‐binding cassette transporter that acts as a molecular sieve in the cytotrophoblasts (CTs) and syncytiotrophoblasts (ST) of the placental barrier. P‐gp prevents the entry of many different substrates to the foetal compartment, including maternally transported steroids, toxins and xenobiotics, and may in this way affect trophic factors directly as well as indirectly.^11^, ^12^ P‐gp also expressed in the EVT‐like first trimester trophoblast cell line HTR8/SVneo,^13^ suggesting a role in EVT migration. However, less is known about P‐gp function in the CT and EVT. Several lines of evidence suggest that P‐gp plays a functional role in the establishment of a healthy and functional syncytium: (a) P‐gp is abundantly expressed at the microvillous surface membrane of the ST facing the placental intervillous space and at the apical membrane of the CT facing the syncytium^12^; (b) proliferation of villous CT, such as during culture in low tension oxygen, is associated with increased expression of P‐gp^12^; and (c) during syncytial differentiation, villous CT normally exit the cell cycle and fuse with the terminally differentiated ST,^14^ but when fusion is dysregulated, villous pathology is established leading to PE and/or IUGR.^14^, ^15^

In the anchoring cell column of the early placenta, EVT cells transition from a proliferating phenotype to a transiently invasive phenotype upon penetration into the decidua and myometrium. These transitioning cells are tightly regulated: proliferating EVT is EGFR/HER1/ITGA5‐positive; invasive EVT is HER2/ITGA1‐positive.^16^, ^17^ Little is known regarding the differentiation of the EVT invasive phenotype once they are within the decidua during the establishment of the uteroplacental circulation. We do know that invasive EVT penetrate the uterine decidua but go no further than the first third of the myometrium before undergoing a terminal differentiation to become non‐invasive. Various types of EVT are observed in the term placental bed including large single cuboidal or polygonal cells, intramural and endovascular trophoblasts and a number of multinuclear trophoblast giant cells (MGC).^18^ How the latter population forms is not known, although both cell‐cell fusion and endoreduplication of EVT are thought to contribute.^19^, ^20^ That failure to exit the invasive pathway is associated with both placenta accreta and choriocarcinoma^21^, ^22^ underlying the importance of placental molecular and cellular integrity in healthy pregnancy outcome.

Based on the function of P‐gp as a molecular sieve, its widespread and abundant expression in CTs and EVTs, and its role in the establishment of a healthy/functional syncytium, we have been suggested that P‐gp regulates CT fate and EVT invasion, which are paramount to placental development, uterine spiral artery remodelling and the establishment of normal maternal‐foetal circulation. We used human placental tissues, the human HTR8/SV_neo_ cell line and two different human placental explant models to conduct our studies. The HTR8/SV_neo_ cell line is widely used for first trimester EVT studies and expresses P‐gp abundantly,^13^ whereas the two ex vivo first trimester placental explant systems model the “EVT pathway” and the “villous pathway”.^14^, ^16^

## 2 MATERIALS AND METHODS

### 2.1 Cell line and culture

HTR8/SV_neo_ is a non‐tumorigenic, invasive SV40 large T‐antigen‐immortalized EVT cell line established in 1993.^23^ Cells were cultured in RPMI 1640 medium (Thermo Fisher Scientific, Canada) supplemented with 10% foetal bovine serum (FBS; Wisent, Canada) and 0.1% Normocin (InvivoGen, San Diego, USA) in a humidified atmosphere containing 5% CO_2_ at 37°C. Cells were confirmed to be free of mycoplasma by PCR. Immunostaining showed that these cells were cytokeratin 8‐positive, while immunonegative for the fibroblast‐specific marker CD90 (Figure S1C).

### 2.2 Tissue collection

First and second trimester placentas (5‐8, 10‐12 and 16‐18 weeks) were obtained from elective terminations of pregnancy. Informed written consent was obtained from each patient and collections were approved by both the Morgantaler Clinic and the Mount Sinai Hospital's Review Committee on the Use of Human Subjects (REB 12‐0007E). Tissue was collected in ice‐cold PBS for villous explant or decidual cell culture. Deidentified frozen placental villous tissue and paraffin tissue sections were obtained from the Placental Biobank of Mount Sinai Hospital, Toronto (all clinical information is detailed in Table 1). Placental samples used in this study were from the following sources: (a) Term healthy elective caesarean sections (38‐41 weeks, n = 9); (b) Preterm placenta both with (n = 4) and without (n = 5) confirmed chorioamnionitis (27‐34 weeks, n = 9 total); (c) Severe early‐onset PE with either an average for gestational age baby (AGA) of 28‐34w (n = 9); or with a small for gestational age baby (SGA) of 28‐34w (n = 9). Frozen tissue was ground under liquid nitrogen in a mortar and pestle and matched sets of RNA and protein were extracted. All research using human tissues was performed in a class II certified laboratory and in accordance with Health Canada guidelines and regulations.

**Table 1:** Clinical and demographic patient data

Demographic	Pre‐eclamptic with AGA baby (n = 9)	Pre‐eclamptic with SGA baby (n = 9)	PTL controls (n = 9)	Term controls (n = 9)
Age (years)				
Mean	31.13	32.52	29.86	32.88
Range	19‐43	20‐42	17‐40	29‐39
Parity				
Nullipara	7	4	6	2^a^
Multipara	2	5	3	7^a^
Gestational age at delivery (weeks)				
Mean	30.4	30.3	29.55	38.6^a^
Range	26.6‐33.2	27.0‐33.0	26.0‐32.6	37.4‐40.1
Indication(s) for delivery				
Severe pre‐eclampsia	9	9	0	0
Non‐reassuring foetal testing	3	9	2	0
Sponta_neo_us preterm labour	0	0	9	0
Induction of labour	4	3	0	0
Caesarean Delivery	9	9	4	9
Max sBP	158.8 ± 18.44^a^	165.2 ± 15.88^a^	122.5 ± 17.89	116.6 ± 11.93
Max dBP	100.7 ± 7.34^a^	107.8 ± 8.91^a^	69.9 ± 11.86	71 ± 7.65
Proteinuria (dip stick)	3‐4	1‐4	n/a	n/a
AST (μ/L)	14‐126^a^	18‐158^a^	10‐22	n/a
ALT (μ/L)	7‐98^a^	15‐129^a^	10‐20	n/a
Creatinine (μmol/L)	50‐102^a^	50‐80^a^	36‐39	n/a
Pre‐pregnancy BMI	28.89 ± 4.05	28.58 ± 5.37	24.10 ± 4.26	26.36 ± 6.64
Celestone treatment	6	9	9	0^a^
Umbilical artery Doppler				
AEDF	3	7^a^	n/a	n/a
REDF	5	2	n/a	n/a
Birthweight (g)	1284.11 ± 304.7	930.0 ± 134.16	1452.54 ± 476.32	3316.25 ± 365.70
Birthweight percentile				
<10th	0	3^a^	0	0
<5th	0	6^a^	0	0
Gender				
Male	3	4	8	5
Female	6	5	1	4
Placental weight (g)	321.0 ± 78.28.	232.67 ± 98.92	362.5 ± 69.07	598.75 ± 107.32^a^

All clinical data were collected from the patient records by the Placental Biobank of Mount Sinai Hospital, Toronto, with ethical approval from the Mount Sinai Research Ethics Board and written patient consent. Statistical differences between the groups were tested by ANOVA analysis or by Student's t test where appropriate.

AEDF, absent end‐diastolic flow; AGA, average weight for gestational age; AST, aspartate transaminase; ALT, alanine aminotransferase; dBP, diastolic blood pressure; SGA, small weight for gestational age; sBP, systolic blood pressure; REDF, reduced end‐diastolic flow.

a *P* < 0.05.

### 2.3 In situ hybridization

Specific sequences for *ABCB1* transporter transcripts were amplified from 8w human placental cDNA samples by PrimeSTAR GXL Polymerase PCR Supermix (Takara Bio, Mountain View, CA, USA) using primers complementary to the reference sequence NM_000927.4: *ABCB1*‐for 5′‐CAG TGT GCT GGA ATT CTT GGC AAA GCT GGA GAG ATC CTC‐3′ with *ABCB1*‐rev, 5′‐GAT ATC TGC AGA ATT CTG TAT CCA GAG CTG ACG TGG C‐3′. After purification, PCR fragments were recombined into an *EcoRI*‐linearized pCRII vector (Thermo Fisher Scientific, Mississaugua, ON, Canada) and transformed into competent Stellar™ *E. coli* cells using the In‐Fusion HD Cloning Kit (Takara Bio). Cloned fragments were verified for sense and antisense orientation by sequencing. For generation of digoxigenin (DIG)‐labelled RNA probes, vectors were linearized (*ABCB1* antisense: *EcoRV*, sense: *BamHI*), phenol:chloroform purified and in vitro transcribed using the T7 High Yield Transcription Kit (Thermo Fisher Scientific) or the SP6 DIG RNA labelling kit (Roche, Canada). Labelled RNA probes were purified using the RNeasy Micro Kit (Qiagen, Canada), and labelling efficiency was analysed by dot blot analysis. In situ hybridizations were performed as described^24^ on 8 μm sections of 4% paraformaldehyde fixed, paraffin embedded placental tissues. Amounts of labelled probes were optimized for each probe preparation.

### 2.4 RNA extraction

Total RNA was extracted using TRIzol (Thermo Fisher Scientific), following a slightly modified protocol for improved yield and integrity.^25^ RNA pellets were resuspended in Millipore water and temporarily stored at −80°C. RNA was DNase I‐treated (1U DNase I per μg of RNA; Thermo Fisher Scientific) and repurified using RNeasy columns (Qiagen). RNA quantity and purity were assessed using NanoDrop ND 1000 spectrophotometry and 1% gel electrophoresis.

### 2.5 Reverse transcription and real‐time SYBR green PCR analysis

RNA samples for subsequent RT‐PCR (1 μg) were reverse transcribed using iScript gDNA clear cDNA synthesis kit (Bio‐Rad, Mississauga, Ontario, CA). cDNA (~20 ng) was used to amplify the reference genes (*YWHAZ* and *TOP1*) or the target genes’ (*ABCB1, ABCC2, ABCG2, HLA‐G, PCNA, GCM1, GJA,1, ERVW‐1 and ASCT‐2)* amplicons using previously optimized or published primers with close to 100% efficiency (Table 2). The reaction mixture was composed of: 3 μL cDNA; 0.8 μL each, 3 μmol/L forward and reverse primers; 0.9 μL RNase/DNase‐free water and 3.3 μL of 2X SYBR green buffer (Millipore Sigma), for a final total reaction volume of 8 μL. Quantitative real‐time (qPCR) using a Bio‐Rad CFX394 real‐time system and accompanying software were used to quantify steady‐state mRNA levels. The qPCR conditions were the following: Heat activation of Taq and denaturation 95°C for 2 minutes and 40 cycles of amplification at 95°C for 10 seconds and 60°C for 30 seconds. Melting curves were generated to confirm the presence of a single band of the expected melting temperature, previously validated by electrophoresis. Experiments were performed in triplicate, technological qPCR replicates were performed in duplicate. All genes were detected at a cT <32, and data were analysed using the 2^−ΔΔCT^ method.

**Table 2:** Real‐time qPCR SYBR green primer sequences

mRNA	Primer name	Sequence
*ABCB1* 30	*ABCB1*‐F	AGCAGAGGCCGCTGTTCGTT
	*ABCB1*‐R	CCATTCCGACCTCGCGCTCC
*ABCC2*	*ABCC2*‐F	GGGCTGCTTTATTTCTTGAGGG
	*ABCC2*‐R	GATGCCTGCTCTTGCTCCTT
*ABCG2* 30	*ABCG2*‐F	TGGAATCCAGAACAGAGCTGGGGT
	*ABCG2*‐R	AGAGTTCCACGGCTGAAACACTGC
*HLAG* 62	*HLAG*‐F	TGCTGAGATGGAAGCAGTCTTC
	*HLAG*‐R	ACTACAGCTGCAAGGACAACCA
*PCNA*	*PCNA*‐F	TTGAAGCACCAAACCAGGAGA
	*PCNA*‐R	TGCAAATTCACCAGAAGGCA
*GCM1* 16	*GCM1*‐F	GGCACGACGGACGCTTTAT
	*GCM1*‐R	GCTCTTCTTGCCTCAGCTTCTAA
*GJA1* 29	*GJA1*‐F	GGGGGATCCATGGGTGACTCGAGC
	*GJA1*‐R	GGGAAGCTTCTAGATCTCCAGGTCATA
*ERVW‐1*	*ERVW‐1*F	AGAAAAGGCCCCAAGAGGTAATAAAG
	*ERVW‐1*R	CCTGGAAAGCAGGGCTATTG
*ASCT‐2* 29	*ASCT‐2*F	CCGCTTCTTCAACTCCTTCAA
	ASCT‐2R	ACCCACATCCTCCATCTCCA
*YWHAZ*	*YWHAZ‐F*	ACTTTTGGTACATTGTGGCTTCAA
	YWHAZ‐R	CCGCCAGGACAAACCAGTAT
*TOP1*	*TOP1‐F*	GATGAACCTGAAGATGATGGC
	*TOP1‐R*	TCAGCATCATCCTCATCTCG

All primers were blasted using NCBI primer blast to ensure specificity to the gene of interest and confirmed to produce a single band of the expected size by PCR analysis.

### 2.6 Western blot analysis

Equal amounts (50 μg) of total placental proteins extracted in lysis buffer (1 mol/L Tris‐HCL pH 6.8, 2% SDS, 10% glycerol containing protease and phosphatase inhibitor cocktail; Thermo Fisher Scientific) were analysed under reducing conditions by Western blot. Proteins were run on a 12% SDS‐PAGE gel and transferred to a PVDF membrane (Millipore, Etobicoke, ON, Canada) at 4°C overnight (wet transfer for high weight bands). Membranes were blocked with 10% non‐fat milk and 0.1% BSA in Tris‐buffered saline with 0.1% Tween (TBS‐T) for 1 hour at room temperature, followed by TBS‐T washes (3X). Membranes were incubated with primary rabbit monoclonal anti‐MDR1 (P‐gp) antibody (1:1000 dilution) (Abcam, Canada) in 5% milk overnight at 4°C followed by 3 washes and a 1 hour incubation with anti‐rabbit horseradish peroxidase (1:3000) linked secondary antibody (GE Healthcare, Canada) at room temperature. Antibody reactions were detected using Bio‐Rad Western C chemiluminescence detection kit (Bio‐Rad), followed by detection of chemiluminescence using a Chemidoc MP gel documentation system (Bio‐Rad). To ensure equal loading, the gel membranes were stripped and reprobed using a rabbit polyclonal antibody to 14,3,3 zeta dilution of 1:2000. We have previously shown that this protein is stably expressed in the human placenta across gestation and in all pathologies.^26^ Band intensity and area were analysed using Quantity One 4.6.7 software (Bio‐Rad).

### 2.7 *ABCB1* siRNA duplex knockdown of HTR8/SV_neo_ cells

Approximately 1.5 × 10^6^ HTR8/SV_neo_ cells in log phase of growth were seeded in 100 mm dishes and grown to 50% confluence over 24 hours. The cells were washed and incubated in medium Optimem (Thermo Fisher Scientific) for 1 hours prior to transfection. Experiments to determine the optimal predesigned siRNA (out of 10) identified 40 nmol/L Dsi2 (specifically targeting exon 10 of the *ABCB1* mRNA) to knockdown *ABCB1* mRNA steady‐state levels maximally over a 48 hours period as compared to the NC‐1 universal control (Integrated DNA Technologies; Canada). Thus, 40 nmol/L Dsi2 *siABCB1* or NC‐1 control was used in all following experiments. siRNA and Lipofectamine RNAiMax (Thermo Fisher Scientific) were first diluted separately in Optimem, then mixed together and incubated at room temperature (RT) for 5 minutes before addition to the cells/placental explants and a 4 hours incubation in a humidified atmosphere containing 5% CO_2_ at 37°C. The supernatant was then removed, the cells/explants were rinsed and fresh RPMI 10% FBS was added and incubated for the experimental period.

### 2.8 Cell growth, viability and fusion of HTR8/SV_neo_ cells

1.5 × 10^6^ HTR8/SV_neo_ cells in log phase of growth were seeded in 100 mm dishes and grown to 30% to 50% confluence over 24 hours. Following transfection with either NC‐1 or Dsi2 *ABCB1,* cells were incubated for a further 24 hours. Cells were counted using the Trypan Blue exclusion method. Cells were first dissociated using Accutase as per the manufacture's protocol (Sigma Aldrich Canada, Oakville, Ontario, Canada). Numbers of viable (clear) and dead (blue) cells were counted using a haemocytometer. In the fusion assays, transfected HTR8/SV_neo_ cells were seeded in 12‐well plates at a density of 7 × 10^5^ cells/well and cultured for a further 48 hours before fixation in 4% PFA and processing for E‐cadherin immunofluorescent staining.

### 2.9 HTR8/SV_neo_ invasion and migration assays

Invasion assays were performed as previously described^27^ using 8 μm (diameter) pore “cell culture inserts” (Corning, Tewksbury, Massachusetts), pre‐coated with growth factor reduced Matrigel (Corning) 0.1 μg/mL in RPMI medium. Matrigel‐coated inserts were rehydrated for 2 hours prior to the experiments. NC‐1 and Dsi2 *siABCB1*‐treated HTR8/SV_neo_ cells were pre‐treated with Mitomycin C (10 μg/mL; Sigma Aldrich, Canada) for 2 hours and then cells were seeded (8 × 10^4^) onto the Matrigel‐coated inserts and incubated for 24 hours in a humidified atmosphere (5% CO_2_ at 37°C). Inserts were fixed in paraformaldehyde (4%; VWR) and stained using the standard haematoxylin and eosin staining protocol. Cells on the non‐invading side of the inserts were removed using cotton swabs moistened with HBSS. Four random fields were then counted, one per quadrant, using a 10 mm grid reticle under a light microscope (100× magnification).

### 2.10 HTR8/SV_neo_ tube formation assay

HTR8/SV_neo_ cells were transfected as above and seeded in Matrigel‐coated 24‐well plates with 5 × 10^4^ cells per well. Cells were incubated overnight at 5% CO_2,_ 37°C. Following incubation (16 hours), three random photographs/well were captured using a DMIL LED inverted microscope (Leica) and micropublisher camera 5.0 RTV (Q Imaging). Total tube length was determined per image (3/well) using Angiogenesis analyser software from Image J http://image.bio.methods.free.fr/ImageJ/?Angiogenesis-Analyzer-for-ImageJ.

### 2.11 Placental explant cultures and treatment

Two ex vivo placental explant models were used to investigate whether P‐gp is involved in EVT and CT differentiation pathways as follows:

#### 2.11.1 EVT pathway

EVT explant cultures were established from first trimester human placentas using the method described previously.^16^ Briefly, placental villi from 5‐8w gestation placentas with pre‐existing anchoring EVT cell columns were microdissected from the placenta and placed on Millicell‐CM culture dish inserts (pore size 0.4 μm; Millipore Corp, Maryland), pre‐coated with undiluted phenol red‐free Matrigel substrate (0.2 mL; Becton Dickinson, Mississauga, Ontario, Canada). Inserts were cultured under a humidified environment at 3% O_2_/5% CO_2_ and 37°C overnight prior to addition of serum‐free DMEM‐Ham's F‐12 media (200 μL; Invitrogen, Canada) supplemented with 1% Normocin (Invivogen), pH 7.4 (Explant Media SFM). EVT outgrowths were established over 48 hours‐culture at 3% ambient oxygen and randomized to treatment group. For *ABCB1* knockdown experimental treatments, SFM was supplemented with the NC‐1 or Dsi2 *siABCB1* transfection reagents (40 nmol/L) and added to the placental EVT explants followed by 48 hours of culture. EVT outgrowth morphology was visually assessed every 24 hours and photographs captured using the DMIL LED inverted microscope (Leica, Concord, ON, Canada) and micropublisher camera 5.0 RTV (Q Imaging).

#### 2.11.2 Villous pathway

Individual clusters of 10‐12 weeks gestation villi were dissected in sterile cold PBS and floated in serum‐free media (DMEM/F12) with 1% liquid media supplement ITS‐A (Sigma, St Louis, MO), 1% Normocin.^14^ These floating villous explants were cultured with intact ST and maintained in 8% ambient oxygen (40 mm Hg) appropriate to the physiological levels of O_2_ in the intervillous space at this gestational time. To investigate the role of P‐gp in CT differentiation, explants were cultured in the presence of NC‐1 or Dsi2 *siABCB1* (40 nmol/L). CT proliferation status was monitored by immunohistochemistry for BrDU (10 mmol/L) incorporation, performed according to the manufacturer's instruction (Thermo Fisher).

Both EVT and fusion experiments were performed on 6 placentas, and explants from each placenta were cultured in triplicate for each treatment point. Explants from both EVT and villous experiments were fixed, wax embedded and sectioned (5 μm) for H&E staining to assess morphology throughout the duration of the experiments (0, 24 and 48 hours). Morphology was assessed by two independent investigators, CED and JJP blinded to treatment group.

### 2.12 Fluorescent immunohistochemistry

**Figure 1:**
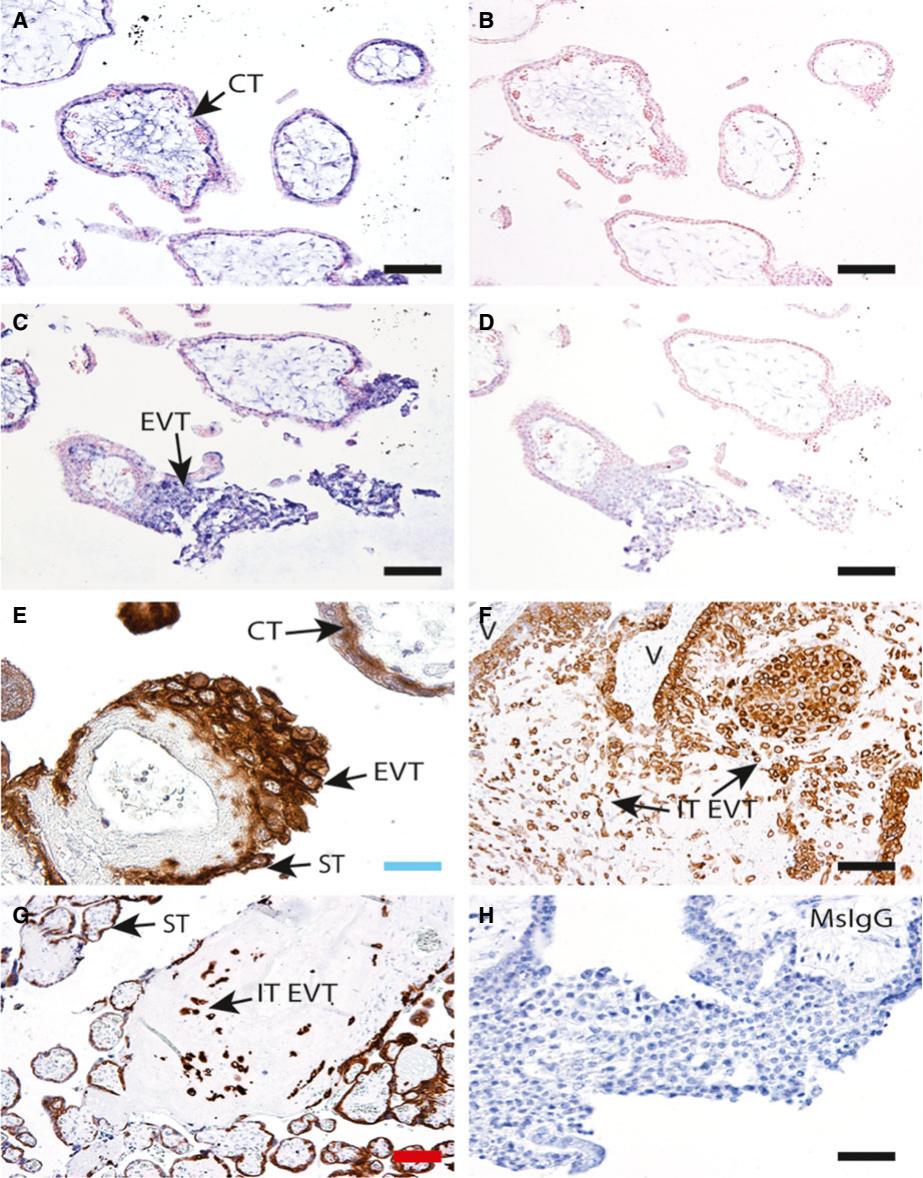
*ABCB1* mRNA and protein localization in CT and EVT. A and C, Representative images of first trimester placental sections showing specific *ABCB1* antisense RNA (probe) hybridization to cytotrophoblast (CT) and extravillous trophoblast (EVT) columns (arrows). B and D, Sense controls in serial sections showing no staining. Representative immunohistochemistry (IHC) showing P‐gp protein localization in E, first trimester; F, 18w decidua basalis; and G, term placental bed and placenta. P‐gp staining is detected in syncytiotrophoblast (ST), CT and EVT in the first trimester and in interstitial EVT (IT‐EVT) within the decidua across gestation (arrows), (V = villous). H, Mouse IgG control. n = 4 placenta at each time‐point. Scale bars: blue = 25 μm, black = 50 μm, red = 100 μm

Placental tissues and explants were fixed and processed to paraffin sections (5 μm) on Superfrost Plus glass slides (VWR, Mississauga, Ontario, Canada). Sections were deparaffinized in xylene and rehydrated through a descending concentration gradient of ethanol. Antigen retrieval specific to each antibody was performed as indicated Table 3. Autofluorescence was reduced using 0.1% Sudan Black in 70% ethanol (1 minutes) and non‐specific binding was blocked using 10% normal goat serum, 2% human serum, 2% donkey serum and 2% rabbit serum for 1 hour.^28^ All primary and secondary antibodies used in these procedures are detailed in Table 3 and were used to assess trophoblast differentiation status (EVT markers: HER2, ITGA1; Syncytial Markers: alpha‐hCG, Cx43 (GJA1) and Syncytin 1 (ERVW‐1)). In the fusion experiments, treated HTR8/SV_neo_ were stained with a rabbit monoclonal antibody against E‐cadherin (Abcam), while in the explant system dual immunohistochemistry (IHC) was performed to visualize the trophoblast populations using either cytokeratin 8/18 (CK8/18) or EVT‐specific HLA‐G staining (Abcam). Primary antibodies were prepared in PBS and were incubated on sections overnight at 4°C. In control experiments, primary antibodies were replaced with mouse and/or rabbit IgG at the same concentration as the primary antibody (Agilent Technologies, Santa Clara, California or Abcam, respectively). Secondary biotinylated antibodies were detected using either Streptavidin‐Alexa488 (1:1000) or Streptavidin‐HRP (1:2000) (Thermo Fisher Scientific); cells were counterstained with the nuclear dye DAPI (5 μg/mL, 1 hour), or as in Figures 1 and 5 developed using DAKO DAB+ system (Agilent Technologies). For the dual staining, either anti‐rabbit or anti‐guinea pig biotin was amplified by Streptavidin Alexa546 (1:1000) (Thermo Fisher Scientific), and markers were visualized by direct secondary staining with anti‐Mouse Alexa488 (1:200) (Abcam). Slides were washed in PBS and mounted in aqueous immuno‐mount (Thermo Fisher Scientific). Immunofluorescent images were captured using images captured from a Quorum Wave FX spinning disc confocal system comprising a Leica DMI 6000B microscope with a Yokoqawa Spinning Head and Image EM Hamematsu EMCCD camera and Velocity imaging software. To ensure fair comparison between control and treatment in the explant experiments, control images were taken first and time of exposure and laser intensity recorded and set. Treatment images were then captured using the same settings for each laser. In the preterm control (PTC) and PE comparison, 5 randomly matched pairs of control and PE placentas were developed in diaminobenzidine (DAB) at the same time and for the same length of time. Fluorescent or DAB intensity was scored semiquantitatively with reference to negative and positive controls on unaltered digital images by two independent investigators blinded to treatment or patient group.^16^

**Table 3:** Antibodies and Conditions

Antibody	Species P = poly M = mono	Source catalog No.	Dilution	Antigen retrieval	Cell type/protein target
Cytokeratin 8/18	Guinea Pig P	Abcamab194130	1:100	Heat: Sodium Citrate buffer pH6	All epithelial cells CT, ST and EVT
HLA‐G (4H84)	Mouse M	Exbio 11‐499‐c100	1:300	Heat: Sodium Citrate buffer pH6	EVT cell specific
Anti‐MDR1 (D11)	Mouse M	Santa Cruz SC‐55510	1:250	Heat: Sodium Citrate buffer pH6	P‐gp protein
Anti‐P‐gp EPR 10364.57	Rabbit M	Abcam ab170904	1:1000	Western Blot	P‐gp protein
Syncytin‐1	Rabbit P	Santa Cruz SC‐50369	1:100	Heat: Sodium Citrate buffer pH6	ST marker
α‐hCG INN hFSH‐132	Mouse M	Acc. Chem. and Sci. Corp. YSRTMCA1026	1:200	Heat: Sodium Citrate buffer pH6	Expressed by ST and found at junction of CT and ST
α‐1 integrin (ITGA1)	Rabbit P	Chemicon AB1934	1:500	Proteinase K, 10 mg/mL 37°C, 10 min	Invasive EVT
c‐erbB2 (HER2)	Rabbit M	DAKO A0485	1:300	Triton 0.01%, 10 min	Invasive EVT and metastatic epithelial breast cancer cells
Cx43	Rabbit P	Invitrogen	1:100	Triton 0.01%, 10 min	Junction of CT and ST

All antibodies used in the study were optimized for antigen retrieval and dilution.

### 2.13 Statistical analysis

Data were assessed for normal distribution. Statistical analyses of data were performed using Prism software on normally distributed data using either the Student's *t* test or a one‐way ANOVA using Bonferroni correction for multiple comparison testing where appropriate. Data are presented as the mean and standard deviation of the data from at least three independent experiments or samples (indicated in the figure legend) performed in triplicate. *P*‐values of <0.05 as compared with respective controls were considered significant (GraphPad Prism; GraphPad Software, San Diego, CA).

Data availability: The data sets generated during and/or analysed during the current study are available from the corresponding author on reasonable request.

## 3 RESULTS

### 3.1 *P‐gp/ABCB1* mRNA and protein expression in the villous and extravillous trophoblast

In situ hybridization (ISH) in first trimester placental sections showed high levels of *P‐gp*/*ABCB1* mRNA in both the villous CT (Figure 1A) and the EVT (Figure 1C). Sense controls did not show any specific staining (Figure 1B,D). Immunohistochemistry using anti‐MDR‐1 antibody (D11) also showed staining in both the villous CT and EVT in the first trimester (Figure 1E, arrows), in the interstitial EVT population within the decidua at 18w (Figure 1F, arrows) and at term (Figure 1G, arrows). Syncytial (ST) staining was also observed in the first trimester (E) and at term. Mouse IgG control showed no specific staining (Figure 1H).

### 3.2 Silencing of P‐gp/*ABCB1* reduces HTR8/SV_neo_ cell proliferation and invasion and induces cell fusion

**Figure 2:**
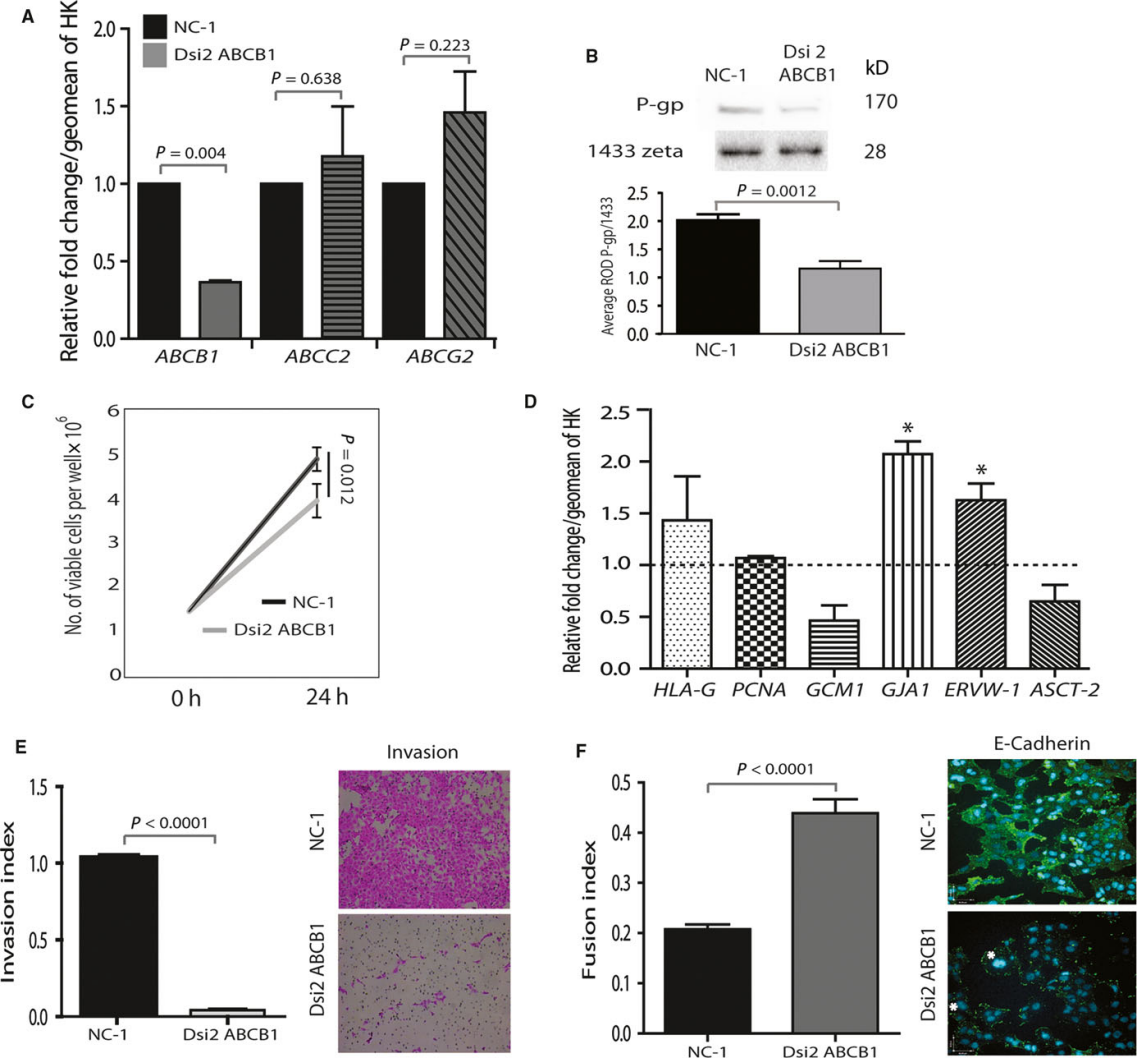
Silencing of *ABCB1* reduced HTR8/SV_neo_ cell proliferation, invasion and induced cell fusion: A, Bar graph representing the relative fold change from the NC‐1 control in average *ABCB1*/*C2* and *G2* mRNA levels assessed by qPCR. *ABCB1* is down‐regulated by 62% (*P* = 0.004), while levels of *ABCC2* and *ABCG2* remain stable. Levels are normalized over the geometric mean of *YWHAZ* and *TOP1* housekeeping genes. B, Representative immunoblot of P‐gp levels in NC‐1 and Dsi2 ABCB1‐silenced HTR‐8 cells. Graph shows the relative band intensity for 170 kDa P‐gp over the housekeeping protein 14,3,3 zeta (*P* = 0.0012). C, Line graph showing the 24‐h growth curves of HTR8/SV_neo_ cells following *ABCB1* knockdown showing a 24% reduction in the proliferative rate in ‐Dsi2 ABCB1 ‐ versus NC‐1 control‐treated cells (*P* = 0.012). D, Bar graph showing the change in EVT and fusion marker mRNA levels 24 h after silencing of ABCB1 as determined by qPCR. Only fusion markers *GJA1* and *ERVW‐1* were increased on treatment with DSi2 ABCB1 (*P < 0.05). Data are expressed as the relative fold change from NC‐1 controls shown by the dashed line. Data were normalized as in A. E and F, Bar graphs representing the HTR8/SV_neo_ invasion and fusion indices following transfection of NC‐1 control or Dsi2 ABCB1. Silencing of ABCB1 results in a > 90% reduction in invasive cells but stimulates a significant two*fold* increase in cell‐cell fusion (*P* < 0.0001). Representative photomicrographs in E show invading cells (located on the bottom of the 8 μm cell inserts) following H&E staining. Photographs in F show a decrease in E‐cadherin staining in *ABCB1* silenced cells and an increase in nuclear size and endomitotic nuclei (*). All experiments were conducted in triplicate on three different passages of HTR8/SV_neo_, and all assessments were performed in duplicate, minimum. Error bars shown in all graphs are the standard deviations from the mean, and data were analysed using one‐way ANOVA and Bonferroni *post hoc* testing or the paired Student's *t* test in D. NC‐1 = universal normal control siRNA duplex. Dsi2 ABCB1 = ABCB1‐specific siRNA duplex

*P‐gp/ABCB1* mRNA was detected by qPCR in HTR8/SV_neo_ cells at cycle values = 28.21 ± 0.91 as compared to first trimester placentas cycle values = 26.19 ± 0.29 (n = 4). Treatment with Dsi2 *siABCB1* knocked down *P‐gp/ABCB1* mRNA levels by 61.7% ± 0.88% (*P* = 0.0004; Figure 2A). Knockdown had no effect on either *ABCC2* or *ABCG2*, two other ATP‐binding cassette transporters of the syncytial barrier (*P* = 0.638 and 0.223, respectively), confirming specificity.

Western blotting, using a rabbit anti‐P‐gp/ABCB1 antibody previously validated in our laboratory,^29^, ^30^ confirmed reduction in P‐gp protein (*P* = 0.0012; Figure 2B). Treatment with Dsi2 *siABCB1* resulted in a concomitant reduction in the number of viable cells (24%) as compared to the NC‐1 control (*P* = 0.012; Figure 2C). Marker gene expression analysis demonstrated that although EVT marker HLA‐G expression was unaffected, silencing of *P‐gp/ABCB1* resulted in the induction of the fusion markers *GJA1* and *ERVW‐1* (*P* < 0.05). Levels of Proliferating Cell Nuclear Antigen (*PCNA*), Glial Cell Missing‐1 (*GCM1)* and Syncytin Receptor (*ASCT2)* were unaffected (Figure 2D).

For migration and invasion assays, HTR8/SV_neo_ cells were pre‐treated with the proliferation inhibitor Mitomycin C prior to invasion assays in order to exclude any proliferative advantage the control NC‐1 might have compared to Dsi2 *siABCB1*. *P‐gp/ABCB1* knockdown resulted in a 95%±3% reduction in invasion (*P* < 0.0001; Figure 2E). In contrast, E‐cadherin staining (green), a marker of cell‐cell fusion, showed a two*fold* increase in number of fused multinuclear aggregates. Fusion index was assessed by counting the number of nuclei in fused areas not separated by E‐cadherin and dividing by the total number of nuclei per image (*P* < 0.0001; Figure 2F). A consistent increase in nuclear size in Dsi2 *siABCB1* compared to NC‐1 controls was also observed, suggestive of endoreduplication, as well as an increase in the number of MGC with large central nuclear aggregates (asterisk; Figure S1A).

### 3.3 Silencing of *P‐gp/ABCB1* drives EVT differentiation, vasculogenic tubule formation and fusion

**Figure 3:**
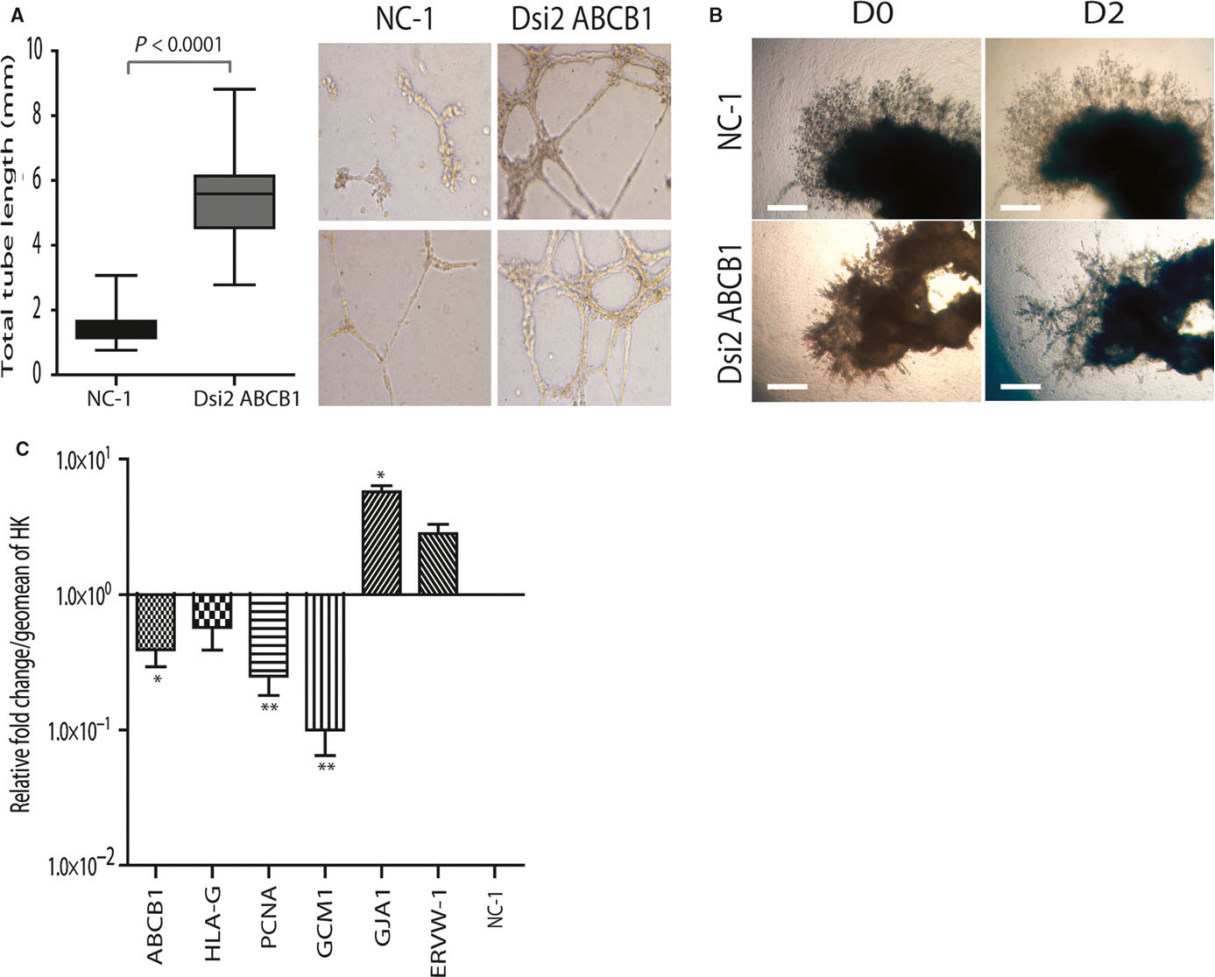
*ABCB1* silencing induces tube formation and fusion markers in primary EVT: A, Representative images of two of three independent tube formation experiments (N1 and N2). HTR8/SV_neo_ cells untreated or transfected with either NC‐1 or Dsi2 *ABCB1* were seeded in Matrigel‐coated plates and incubated overnight. Silencing of *ABCB1* induced a robust and rapid formation of an extensive tubular network as compared to either NC‐1 or untreated controls. Graph shows total tube length over triplicate images per well (*P* < 0.0001 vs NC‐1 control). B, Established EVT outgrowths (5‐7w) were transfected with either NC‐1 (top panels) or Dsi2 *ABCB1* (lower panels) and cultured for 48 h. Explant morphology shows that silencing of *ABCB1* promotes tubule formation in the EVT outgrowth (n = 6). C, Bar graph showing the change in EVT and fusion marker mRNA levels in primary EVT cell columns following 48 h of *ABCB1* silencing as determined by qPCR. Fusion markers *GJA1* and *ERVW‐1* were increased on treatment with DSi2 ABCB1 (*P < 0.05 GJA1 only). *ABCB1* levels decreased as expected (*P* < 0.05) as did *PCNA* and *GCM‐1* (**P < 0.001, n = 3). Data are expressed as the relative fold change from NC‐1 controls at 1.0. Data were normalized as in Figure 2A. Data were analysed using one‐way ANOVA and Bonferroni *post hoc* testing or the paired Student's *t* test in C. Graphs show the mean and standard deviation. Scale bars = 400 μm

HTR8/SV_neo_ cells are well known to spontaneously form tubules when plated on Matrigel.^31^ Upon treatment with Dsi2 *siABCB1*, these cells formed large, extensive networks incorporating the majority of the seeded cells within 16 hours of seeding (Figure 3A). Controls treated with NC‐1 or not treated at all only showed the first stages of early tubule formation at the same time‐point, with most cells remaining as either distinct entities or as small colonies. Quantitation of total tube length showed a highly significant and robust increase above that of controls going from 1.46 ± 0.5 mm in untreated cells to 5.46 ± 1.4 mm in Dsi2‐treated cells (*P* < 0.0001; Figure 3A), representing nearly a three*fold* increase.

In the EVT explant model, we observed an unexpected and previously unreported change in outgrowth morphology. Established EVT outgrowths (D0) were treated with either control (NC‐1) or test (Dsi2 *siABCB1*) for 48 hours (D2), photographed at each time‐point and assessed for changes in outgrowth area and extension. Although there was no overall change in either (data not shown), Dsi2 *siABCB1*‐treated outgrowths formed organized tubes or spikes *within* the Matrigel (not on the surface) as compared to controls that maintained an extended and contiguous outgrowths *on* the surface of the Matrigel (not penetrating within it) (Figure 3B). Trophoblast marker expression also differed. QPCR analysis showed that in microdissected HLA‐G‐positive EVT columns, treatment with DSi2 *siABCB1* resulted in the down‐regulation of P‐gp/*ABCB1* (*P* < 0.05), *PCNA* and *GCM1* mRNA (*P* < 0.01) and in the induction of *GJA1 mRNA* (*P* < 0.05). A trend towards increased *ERVW‐1* (*P* = 0.06; Figure 3C) was also noted.

**Figure 4:**
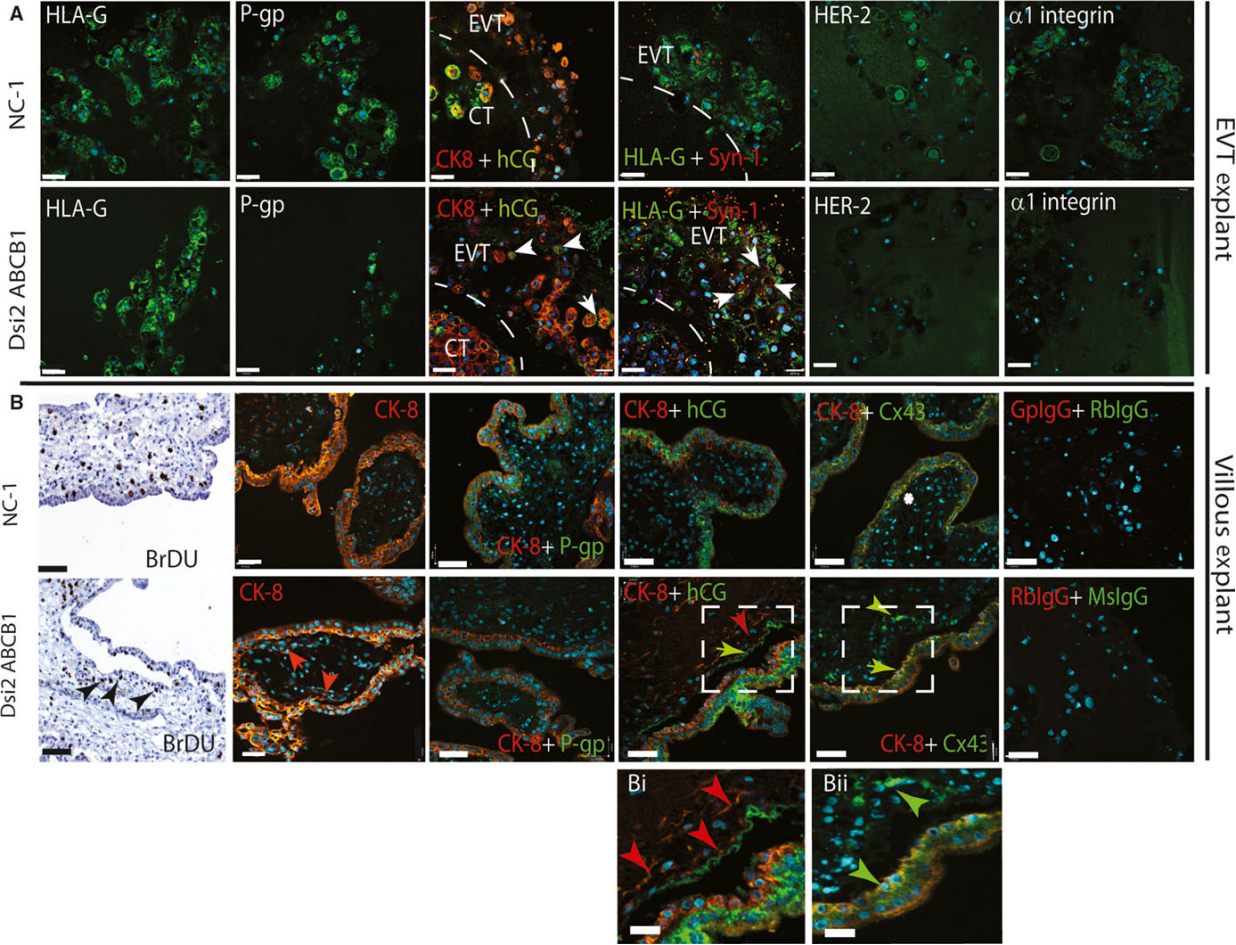
Silencing of *ABCB1* drives EVT and CT syncytial differentiation. A, Fluorescent and dual immunochemistry in serial sections shows the green HLAG‐positive EVT (or red CK8 in the hCG image) in combination with a panel of ST markers (hCG and Syncytin‐1) and EVT markers (HER2 and ITGA1). NC‐1‐treated EVT expresses both HER2 and ITGA1 but not hCG or Syncytin‐1, while Dsi2 *ABCB1*‐treated EVT up‐regulates hCG and Syncytin‐1 but does not express HER2 or ITGA1. B, Floating villous explants (10‐14w) were treated for 24 h with NC‐1 or Dsi2 *ABCB1*. Trophoblast proliferation was assessed by BrDU incorporation and trophoblast fusion by dual fluorescent IHC for cytokeratin CK8/18 (red) and P‐gp, Cx43 and hCG expression (green). In NC‐1 control explants, P‐gp (green) is expressed at the ST with staining extended to the apical CT surface, hCG (green) is expressed at the junction of CT and ST, and Cx43 (green) expression is limited to sites of CT fusion (asterisk). Silencing of *ABCB1* results in separation of the ST, but residual BrDU‐positive CT remains on the villous surface in both CT and newly formed syncytium (black arrows). Formation of a new ST layer on the villous surface can also be observed in the CK8 image (red arrows). P‐gp staining is lost, while both hCG and Cx43 (green arrows) are induced in both the detached ST and newly forming CK‐positive CT‐ST. Magnified images shown in Bi and Bii correspond to the hatched boxes. Negative isotype controls of guinea pig and rabbit IgG or rabbit and mouse IgG showed no staining. Three placentas were used in experimental triplicates. Scale bars = 25 μmol/L and 100 μmol/L in Bi and Bii

Similarly, in the placental explant models, dual immunohistochemistry of EVT outgrowths (Figure 4A) showed that NC‐1‐treated EVT (top panel, marked by HLA‐G staining) expressed P‐gp/ABCB1 and typical markers of invasive EVT (HER2 and ITGA1), but did not express fusion markers hCG or ERVW‐1. Conversely, *siABCB1*‐treated EVT (lower panel), although still expressing HLA‐G, expressed lower levels of P‐gp/ABCB1, HER2 and ITGA1 and increased levels of hCG and ERVW‐1 (white arrows).

In the floating villous explant model (Figure 4B), NC‐1 control explants (top panel) demonstrated an intact cytokeratin‐positive (red) ST layer contiguous with the underlying CT. Viability of the tissue and active CT doubling was demonstrated by the incorporation of BrDU in some cells over 24 hours of culture. As expected, no ST nuclei were BrDU‐positive. Furthermore, P‐gp/ABCB1 was expressed in both CT and ST, and hCG was expressed at the CT‐ST junction. GJA1 localized to the apical surface of the CT in contact with the ST (white asterisk; Figure 4B, top second photomicrograph from right). Silencing of P‐gp/*ABCB1* in contrast (lower panel), led to accelerated CT‐ST fusion and separation of the syncytial layer containing a double row of nuclei. BrDU‐positive cells were observed in both the detached trophoblast layer and the residual CT and newly formed syncytium (black arrows, bottom left image) on the villous surface. This was confirmed by cytokeratin‐8 (CK8) staining as was the formation of a new syncytium (red arrows). P‐gp/ABCB1 expression (green) was undetectable in the Dsi2‐treated explants, confirming knockdown. Dual immunofluorescence combining CK8 staining with hCG and GJA1 showed that hCG (green arrow) localized to the apical surface of the new ST (red arrow), while de novo expression of GJA1 was induced at the junction between CT and ST (green arrow). Magnified areas are indicated in the hatched boxes. Both hCG and GJA1 levels increased in the detached syncytial layer.

### 3.4 P‐gp/ABCB1 expression is decreased in PE placenta

**Figure 5:**
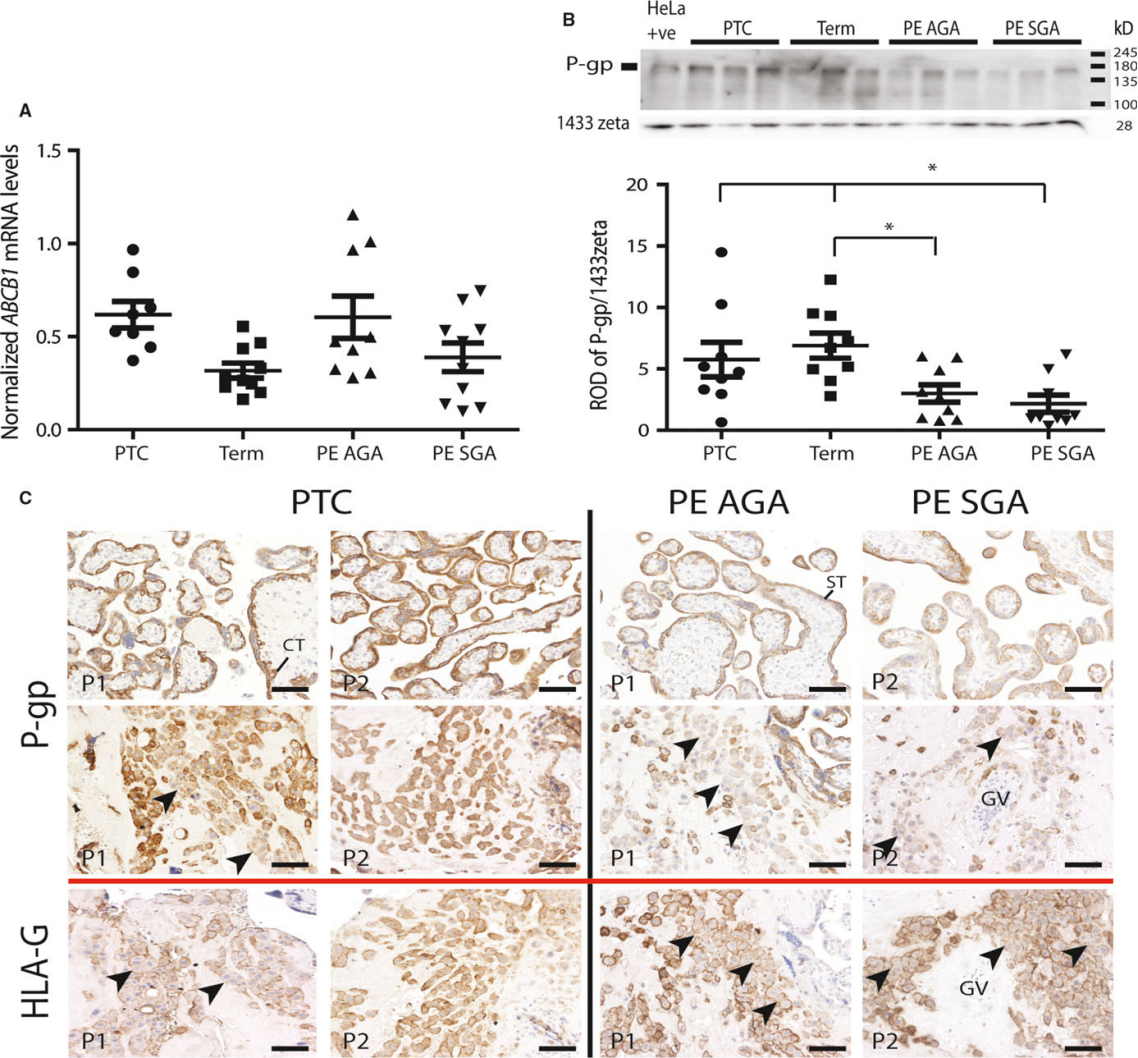
ABCB1/P‐gp is decreased in severe early‐onset PE placentas. A, Real‐time qPCR analysis of ABCB1 mRNA levels in preterm control (PTC), elective Caesarean section term controls (Term) and severe early‐onset pre‐eclamptic placenta (PE) with a normally grown foetus (AGA) and with a growth restricted foetus (SGA). Data are expressed as normalized against the geometric mean of the housekeeping genes TOP1 and YWHAZ. Although one‐way ANOVA identified significant variation among all samples, *post hoc* testing showed no significant difference between groups. B, Representative Western blot of ABCB1 protein expression in the same samples. The anti‐ABCB1 antibody identified a 170 kDa protein corresponding to the HeLa cell positive control. Quantification using the housekeeping protein 14,3,3 zeta (product of the YWHAZ gene) showed that ABCB1 levels were significantly decreased in both PE groups as compared to gestationally matched (PTC) and healthy controls (*P < 0.05, n = 9/group). Error bars shown in all graphs are the standard deviations from the mean, and data were analysed using one‐way ANOVA and Bonferroni *post hoc* testing. C, Representative images of P‐gp immunohistochemical staining in PTC and PE villous placenta and placental bed (2 examples shown for each, P1 and P2). Analysis was undertaken in n = 5/group. PTC placentas show strong CT and ST staining in the placental villi (upper panel) as compared to the PE placentas. EVT in the PTC bed (middle panel) show high levels of P‐gp in isolated EVT. Fewer isolated EVT are observed in the PE placental bed, and the EVT is aggregated showing an increase in areas of cell‐cell fusion (arrows) and expresses a low level of P‐gp as compared to PTC. Fused cells can be visualized in the corresponding serial sections by HLA‐G immunoreactivity (lower panel). Scale bar=25 μm

Although one‐way ANOVA analysis of the qPCR data from *ABCB1* mRNA levels in healthy, preterm and severe PE placentas identified significant variation (*P* < 0.05), subsequent *post hoc* tests did not show any significant difference between patient groups (*P* = 0.0595; Figure 5A).

Western blotting of term and preterm controls and severe early‐onset PE placental samples detected a 170 kDa protein in all samples, which corresponded to the band detected in the positive control HeLa cell lysate (Figure 5B). Quantitation against the 14,3,3 zeta housekeeping protein showed that P‐gp/ABCB1 protein levels were dramatically reduced in PE placentas from pregnancies with a either a normal or a growth restricted baby as compared to gestational‐matched preterm groups (PTC) and to healthy term controls (*P* < 0.05). Immunohistochemistry similarly showed a reduction in P‐gp/ABCB1 protein levels in PE placentas as compared to gestationally matched preterm controls (PTC, Figure 5C). In the villous CT and ST of PTC placenta, P‐gp/ABCB1 staining was consistently strong, whereas, in the PE placentas, which displayed characteristic CT depletion and ST aggregation, P‐gp/ABCB1 was weakly expressed in the syncytial cytoplasm (PE, Figure 5C). In the HLA‐G+ EVT of the placental bed, P‐gp/ABCB1 was also strong in both the membrane and cytoplasm of individual positive EVT but was decreased in the cytoplasm in the fused aggregates of both PE and PTC (black arrows). As expected, the EVT in the PE placental bed were clustered together and showed a shallow invasion of the decidua as compared to PTC. Furthermore, an increased number of MGC were seen in the PE placental bed as compared to the PTC (arrows in HLA‐G sections showing loss of HLA‐G in fused areas). Levels of P‐gp/ABCB1 were consistently lower in EVT in the PE placental bed as compared to PTCs.

## 4 DISCUSSION

In this study, we have shown that P‐gp is expressed at both the mRNA and protein levels in the proliferating CT, in the ST and in the invasive EVT of the first trimester human placenta, as well as in the ST of the term placenta. EVT‐specific expression was maintained in invasive interstitial EVT throughout gestation and in differentiated individual cuboidal EVT at term. These data confirm, extend and validate our previous reports of P‐gp expression in these trophoblast subtypes.^11^, ^19^, ^32^ This is important considering that P‐gp is a critical component of the placental barrier and hence critical for a healthy pregnancy outcome, through its role in maintaining a balance of trophic factors. Notably, these data also further demonstrate a functional role for P‐gp in EVT invasion.

To first investigate this, we utilized the first trimester EVT cell line HTR8/SV_neo_ and studied the effects of silencing *ABCB1* expression in proliferation, invasion and fusion assays. We showed that specific and effective silencing of *ABCB1* reduces the proliferation rate of HTR8/SV_neo_ cells. Furthermore, we demonstrated for the first time, a role for P‐gp in EVT invasion, as silencing of its expression results in the near complete blockade of HTR8/SV_neo_ invasion and migration (Figure S1B). Other studies have shown that P‐gp is essential for immune cell migration; as either verapamil (a calcium channel blocker and inhibitor of P‐gp) or neutralizing antibodies against P‐gp have been shown to prevent the migration of both dendritic cells and T cells out of cultured human skin explants.^33^ More recently, overexpression of P‐gp in breast and oral cancer cell lines has been shown to lead to increased PI3K‐mediated membrane ruffling, the first step of the invasive process, in response to drugs transported by P‐gp.^34^ These studies raise the interesting possibility that P‐gp‐mediated export of trophoblastic factors plays a functional role in active EVT invasion. Testing this is, however, beyond the scope of this first study due to the complexity of the inflammatory early implantation site environment and the many differential effects of progesterone, growth factors, chemokines, sphingolipids and cholines on P‐gp expression and function.^35^ It remains an important question and will be the focus of future studies.

Interestingly, although trophoblast invasion was prevented by *ABCB1* silencing, we showed in HTR8/SV_neo_ cells that knockdown of *ABCB1* induced markers of fusion such as *GJA1* (Cx43), *ERVW‐1* (Syncytin‐1) and *hCG* and increased the fusion index with aggregates showing features of both endoreduplication and endomitosis.^36^ It is known that HTR8/SV_neo_ cells are maintained by a stem cell side population (SP) that expresses a higher level of *ABCG2* mRNA than the non‐side population (NSP).^37^ Moreover, treatment of the SP HTR8/SV_neo_ with verapamil (a P‐gp inhibitor) was similarly shown to result in their terminal differentiation to the NSP subtype.^37^ These results collectively support a potential role for P‐gp in the maintenance of the trophoblast progenitor population.

HTR8/SV_neo_ cells are also well known to spontaneously form tubes when seeded on Matrigel and express genes that are associated with actin cytoskeleton organization, cell migration and blood vessel development.^31^ However, when *ABCB1* was silenced, we observed an increase in the speed of tube formation and a large increase in the complexity and in the length of the total tube network formed compared to that observed in controls. More unexpectedly, we observed a similar effect of *ABCB1* knockdown on vasculogenesis and syncytialization of primary EVT cells in both explants and microdissected EVT anchoring columns. In *ABCB1*‐silenced EVT outgrowths, we reproducibly observed distinct tubes and spikes that, although remaining positive for the EVT marker HLA‐G, coexpressed the syncytial fusion markers hCG and Syncytin‐1 but lost the invasive EVT markers ITGA1 and the HER2 receptor.^16^, ^38^

We also observed an acceleration in CT fusion to form ST in the floating villous explant model where silencing of *ABCB1* resulted in the separation of a double‐layered syncytium with the formation of a new syncytium underneath it, within 24 hours. This process usually takes 72 hours in untreated explants which will shed and spontaneously generate a new syncytium in appropriate culture conditions.^14^, ^39^ In both the separated layer and in the new syncytium, Cx43 levels were induced at the junction between CT and SYT, and hCG was increased at the apical side of the new syncytium, indicating acceleration of CT fusion to form ST. The role of P‐gp in trophoblast fusion is less surprising as a number of studies have reported the loss or down‐regulation of P‐gp in the spontaneous differentiation of primary CT to ST.^40^, ^41^, ^42^ It is also well known that trophoblast fusion stimulated by the protein kinase A pathway and cAMP involves the induction of GJA1 and hCG.^43^ Knockdown of their expression in the BeWo choriocarcinoma model results in an arrest of forskolin‐stimulated fusion,^44^, ^45^ while overexpression of Cx43 permits Jeg‐3 cell fusion.^28^

As a point of speculation, beyond its well‐known role in substrate distribution and bioavailability and in multidrug resistance, P‐gp plays a role in cancer cell invasion, drug resistance and metastasis.^46^, ^47^, ^48^, ^49^ For example, proto‐oncogene tyrosine‐protein kinase SRC‐mediated phosphorylation of Annexin A2 (ANXA2) leads to its direct interaction with P‐gp and promotes breast cancer cell invasion in vitro.^50^ The findings reported here seen from the epithelial‐mesenchymal transition (EMT) perspective, as they are utilized by both tumour cells and trophoblast cells, raised the intriguing possibility that P‐gp can directly regulate EVT differentiation. Our observations may reflect the final or terminal EMT of the IT‐EVT phenotype into MGC. In vivo, invasive interstitial HLA‐G+ EVT (IT‐EVT) within the decidua have been reported to accumulate in clusters preceding their fusion into MGC after 12 weeks of gestation^20^ and to express both ERVW‐1 and hCG.^51^, ^52^ As stated above, this process is purely speculative, as no actual cell fusion event has been visualized in the placental bed and the alternative pathway of endoreduplication is also suggested to contribute to MGC formation.^53^ Collation of the effects of *ABCB1* silencing in our EVT models supports the hypothesis of cell‐cell fusion in MCG formation but also suggests a joint role with endoreduplication and endomitotic events as seen in the HTR8 fusion assay.

Interestingly, we also observed a significant decrease in P‐gp protein levels in severe early‐onset PE villous placenta samples as compared to gestational‐matched preterm controls (PTC). This decrease was only observed at the protein level suggesting that post‐translational regulation of P‐gp may be affected as has been reported in MCF‐7 doxorubicin‐resistant cancer cell lines.^54^ In the IHC, the PE placenta displayed an accelerated ST maturation and loss of CT as expected,^55^ along with a decrease in P‐gp protein, demonstrating diffuse cytoplasmic syncytial and vasculo‐syncytial membrane staining and mirroring our observations in the floating explant model. In contrast, the PTC placenta, like the term placenta in Fig. 1, displays both strong CT and ST vasculo‐syncytial membrane staining.

Pregnancies complicated by pre‐eclampsia (PE) also display an increase in numbers of MGC in the placental bed greater than that of healthy controls.^18^, ^56^ Al‐Nasiry and colleagues have suggested that this increase may be associated with accelerated interstitial IT‐EVT fusion as assessed by loss of E‐cadherin staining.^57^ Interestingly, we found that P‐gp expression was also reduced in the IT‐EVT in the placental bed of severe early‐onset PE placentas as compared to gestational‐matched preterm controls. In PE placental beds, EVT was restricted to the peripheral decidua and predominantly accumulated in clusters containing an increased number of MGC that were weakly P‐gp‐positive. In contrast, in preterm control placental beds, the EVT had invaded far more extensively and lower numbers of fused multinucleate areas with concomitant loss of P‐gp were observed. Endoreduplication events cannot be excluded as the nuclei of these cells are also enlarged. P‐gp staining was strong in isolated individual EVT in the placenta bed in both preterm controls and PE EVT, again supporting a role for P‐gp in maintenance of individual EVT cells. Excessive decidual inflammation during the early events of EVT invasion is a contributory factor in shallow placentation, abruption and PE.^58^ These data, together with our previous data showing that preterm placentas with acute chorioamnionitis also show decreased human placental P‐gp expression^59^ and that acute maternal inflammation leads to decreased placental P‐gp activity in a pregnant mouse model,^60^ lead us to suggest that aberrant early decidual inflammation may reduce P‐gp expression by EVT. This would accelerate EVT terminal differentiation and thus contribute to the failure of EVT‐mediated uterovascular transformation associated with the development of PE.^61^, ^62^ Further investigation of the function of P‐gp in EVT will enhance our understanding of human placentation and disease and of placental epithelial‐mesenchymal transition (EMT) invasion processes in particular. Our data also highlight a need for an investigation of P‐gp expression and function in the invasive pathologies of the placenta, such as choriocarcinoma and placenta accreta.

In summary, we have shown for the first time that functional silencing of P‐gp results in the terminal differentiation of both CT and EVT along the fusogenic syncytial pathway. These results support a key role for *ABCB1* and P‐gp in the maintenance of villous and extravillous trophoblast populations of the placenta. As these pathways of trophoblast turnover and differentiation are compromised in both IUGR and PE placentas, we suggest that P‐gp loss or down‐regulation may contribute to the development of these placental pathologies. It is important to note that, as P‐gp function is affected by a number of prescription drugs and inflammatory events, the implications of a functional role for P‐gp in trophoblast cell fate and placental development are of critical importance to a healthy pregnancy. To our knowledge, this is the first study on the functional role of P‐gp in the EVT and invasive phenotypes.

## AUTHOR CONTRIBUTIONS AND CONFLICT OF INTEREST

CED, JJP, MS and SGM conceived and designed the experiments. CED, JJP, PL and MK performed the experiments and analysed the data. MS, SL and SGM contributed to reagents/materials/analysis tools. CED and JJP wrote the manuscript. CED, JJP, EB, MJ, SL, MS and SGM revised the manuscript. SL, SGM and MS conceptualized the research programme and secured the grant funding. The authors have declared that no conflict of interest exists.
