# Supplementary material for: P‐Glycoprotein (P‐gp)/ABCB1 plays a functional role in extravillous trophoblast (EVT) invasion and is decreased in the pre‐eclamptic placenta
Source: J Cell Mol Med. 2018 Sep 5;22(11):5378–93. doi: 10.1111/jcmm.13810 (PMC6201374; doi:10.1111/jcmm.13810)

P-Glycoprotein (P-gp) maintains villous and extravillous trophoblast populations and is decreased in the preeclamptic placenta

Caroline E. Dunk* Jane J. Pappas, Phetcharawan Lye, Mark Kibschull, Mohsen Javam, Enrrico Bloise, Stephen J. Lye, Moshe Szyf and Stephen G. Matthews.

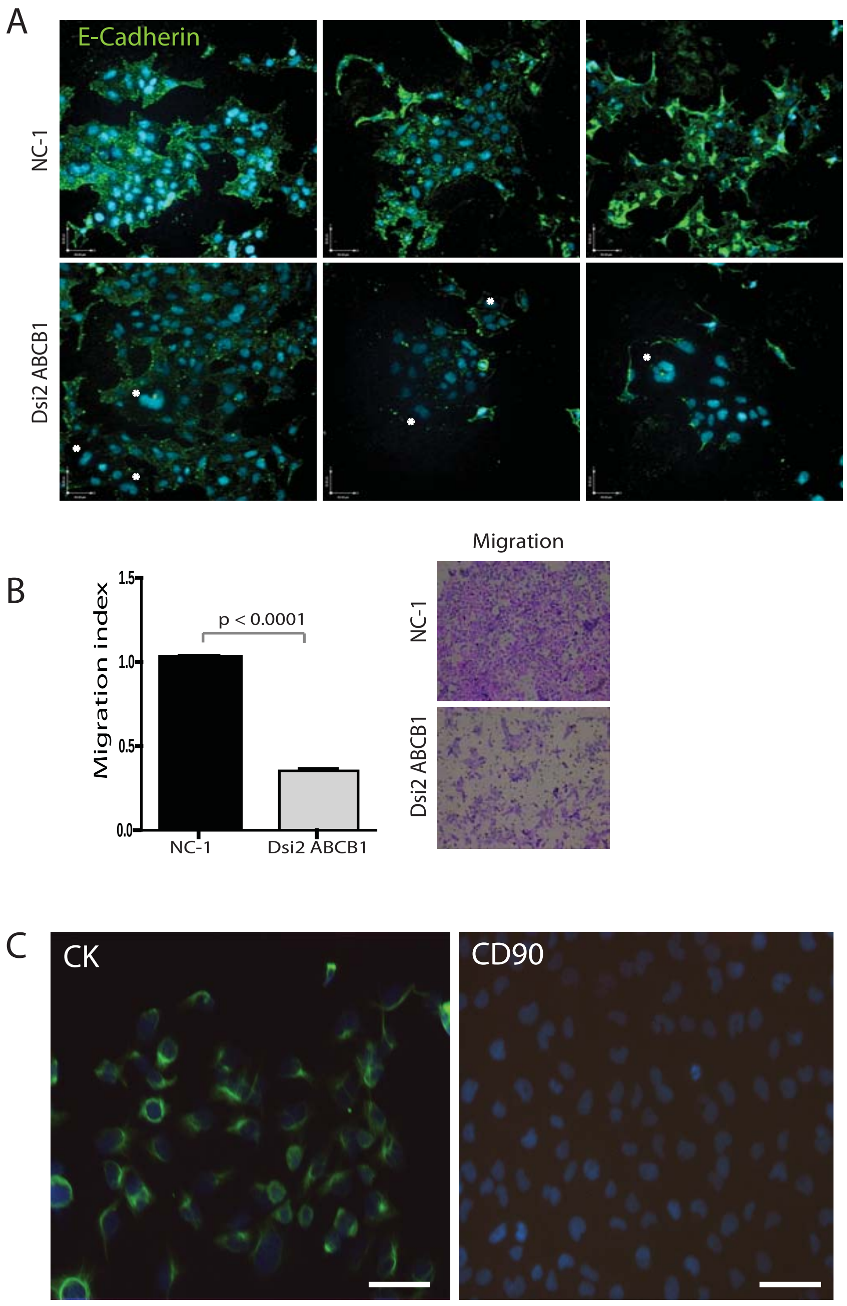

Supplement: Supplementary file 1 [file JCMM-22-5378-s001.pdf]
