# Supplementary figures and images for: P‐Glycoprotein (P‐gp)/ABCB1 plays a functional role in extravillous trophoblast (EVT) invasion and is decreased in the pre‐eclamptic placenta
Source: J Cell Mol Med. 2018 Sep 5;22(11):5378–93. doi: 10.1111/jcmm.13810 (PMC6201374; doi:10.1111/jcmm.13810)

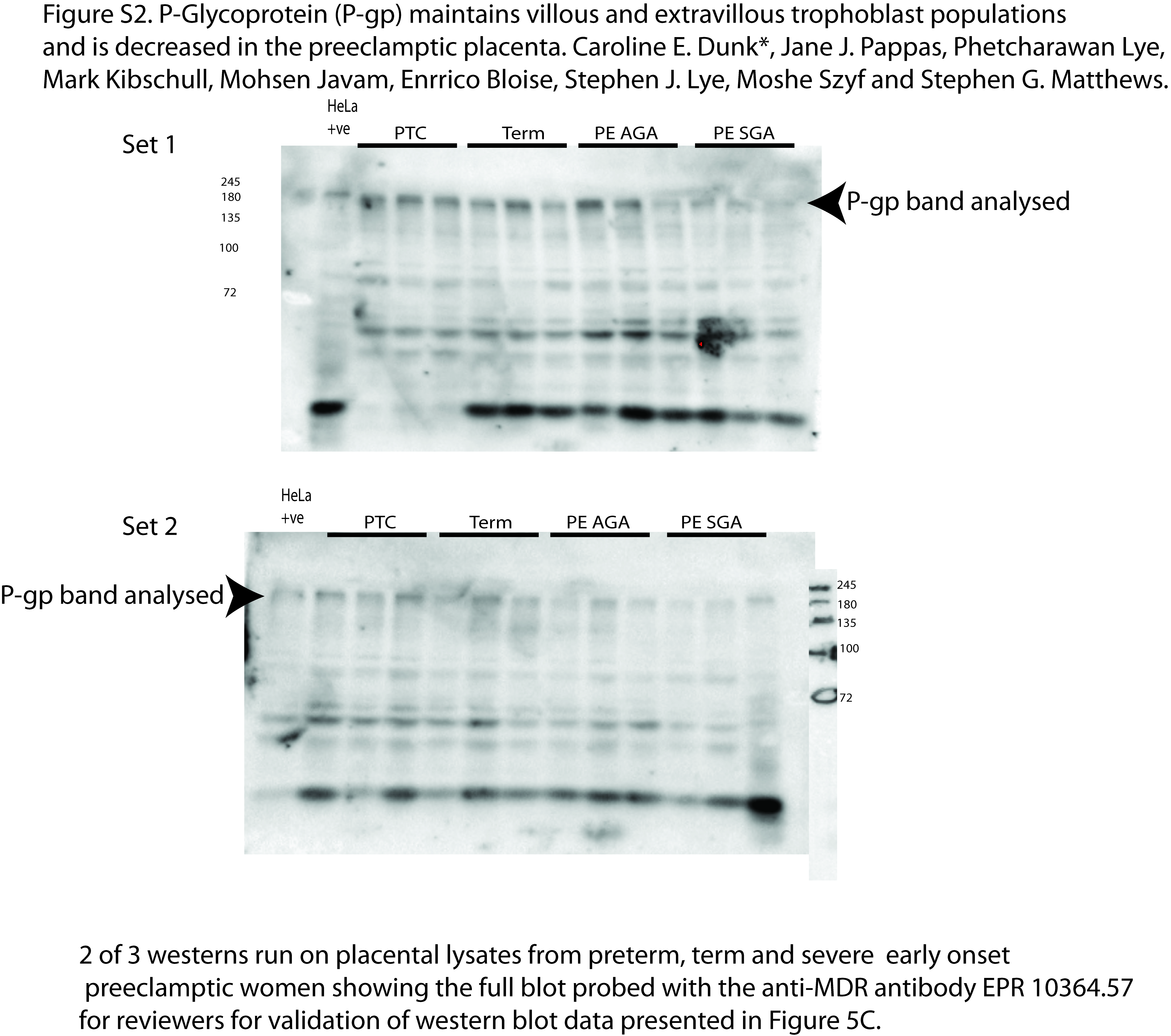

Supplement: Supplementary file 2 [file JCMM-22-5378-s002.tif]
