# Supplementary material for: P‐Glycoprotein (P‐gp)/ABCB1 plays a functional role in extravillous trophoblast (EVT) invasion and is decreased in the pre‐eclamptic placenta
Source: J Cell Mol Med. 2018 Sep 5;22(11):5378–93. doi: 10.1111/jcmm.13810 (PMC6201374; doi:10.1111/jcmm.13810)
Supplement: Supplementary file 3 [file JCMM-22-5378-s003.docx]

**Supplementary Figure S1. A:** Representative fusion images from three independent passages of HTR8/SV_neo_ cells treated with NC-1 universal control siRNA or DSi2 *ABCB1*. All silenced cells show an increase in cell fusion and the appearance of Multinucleated Giant Cells (MGC) accompanied by an increase in endomitotic and endoduplicated large nuclei. **B:** Silencing of ABCB1 significantly inhibits HTR8/SV_neo_ cell migration. Representative photomicrographs show invading cells (located on the bottom of the 8 μm cell inserts) following H&E staining. **C:** HTR8 cells do not express fibroblast marker CD90. In contrast to recent reports stating that HTR8/SV_neo_ cells express Vimentin, and thus are a mix of trophoblast and fibroblast (Abou-Kheir W, Barrak J, Hadadeh O, Daoud G. HTR‐8/SVneo cell line contains a mixed population of cells. Placenta. 2017;50:1‐7. 15), here we show that while all HTR8/SV_neo_ cells express Cytokeratin (CK), none express the fibroblast specific marker CD90. We suggest that alternatively vimentin may be marking the reported stem cell SP population in the HTR8/SV_neo_ line.

**Supplementary Figure S2.** Two of three western blots run on placental lysates from pre-term, term and severe early onset pre-eclamptic women showing the full blot probed with the anti-MDR antibody EPR 10364.57 for reviewers for validation of western blot data presented in Fig. 5C.
